# 2207: A Phase I dose escalation trial of nab-paclitaxel and fixed dose radiation in patients with unresectable or borderline resectable pancreatic cancer

**DOI:** 10.1017/cts.2017.121

**Published:** 2018-05-10

**Authors:** Jacob Ezra Shabason, Jerry Chen, Smith Apisarnthanarax, Nevena Damjanov, Bruce Giantonio, Arturo Loaiza-Bonilla, Peter O’Dwyer, Mark O’Hara, Kim Reiss, Ursina Teitelbaum, Paul Wissel, Jeffery Drebin, Charles Vollmer, Michael Kochman, Rosemarie Mick, Norge Vergara, Nirag Jhala, Abigail Berman, Jay Dorsey, Sydney M. Evans, Gary Kao, John N. Lukens, John P. Plastaras, James M. Metz, Edgar Ben-Josef

**Affiliations:** University of Pennsylvania, Philadelphia, PA, USA

## Abstract

OBJECTIVES/SPECIFIC AIMS: Patients with locally advanced pancreatic cancer typically have poor outcomes, with a median survival of ~16 months. Novel methods to improve local control are needed. Nab-paclitaxel (abraxane) has shown efficacy in pancreatic cancer and is FDA approved for metastatic disease in combination with gemcitabine. Nab-paclitaxel is also a promising radiosensitizer based on laboratory studies, but it has never been clinically tested with definitive radiotherapy for locally advanced disease. METHODS/STUDY POPULATION: We performed a phase 1 study using a 3+3 dose-escalation strategy to determine the safety and tolerability of dose escalated nab-paclitaxel with fractionated radiotherapy for patients with unresectable or borderline resectable pancreatic cancer. Following induction chemotherapy with 2 cycles of nab-paclitaxel and gemcitabine, patients were treated with weekly nab-paclitaxel and daily radiotherapy to a dose of 52.5 Gy in 25 fractions. Final dose-limiting toxicity (DLT) determination was performed at day 65 after the start of radiotherapy. RESULTS/ANTICIPATED RESULTS: Nine patients received nab-paclitaxel at a dose level of either 100 mg/m^2^ (n=3) or 125 mg/m^2^ (n=6). One DLT (grade 3 neuropathy) was observed in a patient who received 125 mg/m^2^ of nab-paclitaxel. Other grade 3 toxicities included fatigue (11%), anemia (11%), and neutropenia (11%). No grade 4 toxicities were observed. With a median follow-up of 8 months (range 5–28 months), median survival was 19 months and median progression-free survival was 10 months. Following chemoradiation, 3 patients underwent surgical resection, all with negative margins and limited tumor viability. Of the 3 patients, 2 initially had borderline resectable tumors and 1 had an unresectable tumor. Tumor (SMAD-4, Caveolin-1) and peripheral (circulating tumor cells and microvesicles) biomarkers were collected and are being analyzed. DISCUSSION/SIGNIFICANCE OF IMPACT: The combination of fractionated radiation and weekly nab-paclitaxel was safe and well tolerated. This regimen represents a potentially promising therapy for patients with unresectable and borderline resectable pancreatic cancer and warrants further investigation.

